# Non-canonical WNT/PCP signalling in cancer: Fzd6 takes centre stage

**DOI:** 10.1038/oncsis.2017.69

**Published:** 2017-07-24

**Authors:** G Corda, A Sala

**Affiliations:** 1 College of Health and Life Sciences, Brunel University London, Uxbridge, UK; 2 Institute of Environment, Health and Societies, Brunel University London, Uxbridge, UK; 3 Dipartimento di Scienze Psicologiche, della Salute e del Territorio, University ‘G d'Annunzio’ Chieti-Pescara, Centro Studi sull'Invecchiamento, Chieti, Italy

## Abstract

Frizzled receptors are the mediators of the wnt canonical and non-canonical pathways, which play fundamental roles in cell differentiation and organism development. A large body of work indicates that dysregulation of wnt signalling is a feature of oncogenic transformation, but most of the studies published so far focus on the assessment of the consequences of aberrations of the canonical pathway in human cancer. In this review, we discuss the emerging role of the wnt non-canonical pathway regulated by frizzled receptor 6 (Fzd6) in the pathogenesis of different types of human malignancies. The function played by Fzd6 in the physiology of normal and cancer cells has been highlighted in the view that an increased knowledge of the signalling pathways upstream and downstream of this receptor could ultimately result in the identification of new targets for cancer therapy.

## Introduction

**Figure 1:**
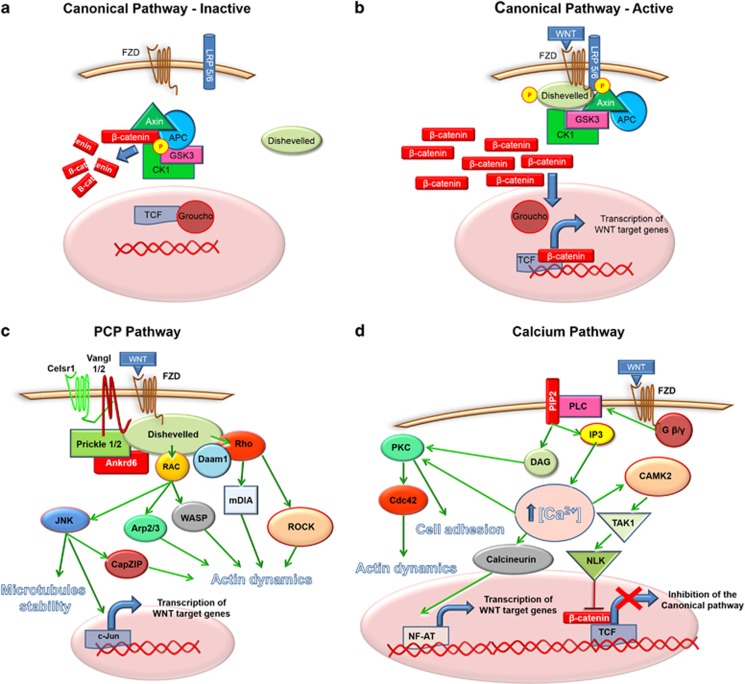
The figure illustrates the wnt canonical (**a**, **b**), non-canonical (**c**, **d**) pathways and the main molecules involved.

Frizzled receptors (FZDs) are seven-transmembrane-spanning proteins belonging to a sub-class of the G protein-coupled receptor family.^1^ In humans, there are 10 FZD receptors each encoded by a separate gene. Interaction of wnt ligands with FZDs results in the activation of the canonical or non-canonical wnt signalling pathways regulating embryonal development, cell proliferation, motility, polarity, stem cell maintenance and tissue differentiation.^2^ The main molecules involved in the canonical and non-canonical pathways are illustrated in Figure 1. Despite recent advances in the understanding of the function of FZDs and wnts, some aspects of the wnt pathway are still poorly understood. This is probably a consequence of the intricate connections between the 19 wnt ligands and the 10 FZDs, making the signalling pathway an intrinsically complicated system to study. Classically, activation of the wnt canonical pathway leads to the stabilization and translocation of β-catenin into the cell nucleus, where it promotes the transcription of wnt-associated genes.^3^ The term non-canonical pathway refers to a group of wnt-dependent signalling pathways which do not lead to the cytoplasmic stabilization of soluble β-catenin. Two of these pathways have been well characterized: the planar cell polarity (PCP) and the wnt-Calcium pathway. Although, for simplicity, wnt signalling is often dichotomized in two branches, the canonical and the non-canonical pathways often overlap to coordinate complex cellular responses.

Nusse and Varmus were the first to observe that MMTV (mouse mammary tumour virus) infection induces mammary tumours in mice through activation of the WNT1proto-oncogene.^4^ Since then, many distinct alterations in the wnt pathway have been related to carcinogenesis and tumour development. Deregulated expression or mutations of FZDs genes have been observed in various human malignancies, indicating the prominent role of these signalling molecules in cancer.^5, 6, 7, 8, 9, 10, 11, 12^

## Physiology of Frizzled receptor 6

**Figure 2:**
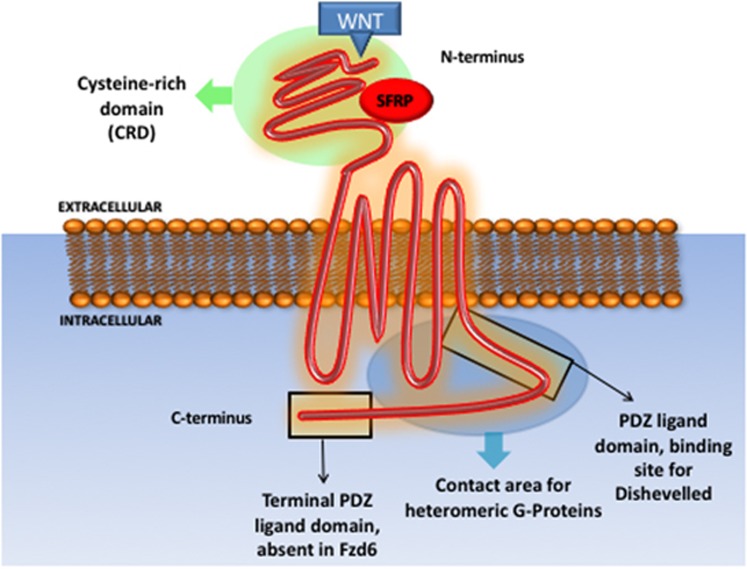
Frizzled receptors domains and interacting proteins.

Frizzled receptor 6 (Fzd6) is a 706 amino acid, 7 transmembrane domain receptor, encoded by the FZD6 gene, located in chromosome 8 (8q22.3-q23.1). Similarly, to other FZD receptors, Fzd6 contains an extracellular cysteine-rich domain, the binding site for wnt proteins^13^ and Soluble Frizzled-related proteins.^14^ In contrast to other members of the Fzd family, Fzd6 does not contain a second C-terminal PDZ domain-binding motif^15^ (Figure 2).

Fzd6 has been shown to regulate both canonical and non-canonical pathways, but most reports indicate a prevalent role in the non-canonical pathway.

Golan and collaborators observed that the ectopic expression of FZD6 in HEK293 cells did not result in the activation of the canonical pathway, even in the presence of canonical ligands. Conversely, the ectopic expression of FZD1 led to a significant activation of the canonical pathway in similar conditions. The group identified Fzd6 as an inhibitor of the canonical pathway through activation of TAK1/NLK kinases, which in turns reduce activation of β-catenin target genes via phosphorylation of TCF/LEF transcription factors.^16^ These early results indicated that Fzd6 could be upstream of the wnt/Calcium pathway. Accordingly, studies on canine kidney epithelial cells demonstrated that Fzd6 physically binds to the canonical ligand wnt4, but ectopic expression of FZD6, together with WNT4, did not induce any greater wnt4-dependent activation of TCF/LEF reporter, suggesting a non-canonical role for Fzd6 in this system.^17^ Interaction of Fzd6 with wnt4 was also observed in murine hematopoietic precursor cells; depletion of Fzd6 abrogates the wnt4 dependent expansion of these cells, suggesting that the receptor is critically required for wnt4 function.^18^ In another study conducted in HelaS3 cells it was reported that downregulation of FZD6 did not change accumulation of nuclear β-catenin, nor the activation of RAC following stimulation with the canonical ligands wnt3a or wnt5a, further suggesting a non-canonical role of the receptor. The same study also revealed that the Fzd6 cysteine-rich domain does not bind to wnt3a and wnt5a.^19^ A FRAP assay was conducted to identify Fzd6 ligands by measuring the membrane shift of Fzd6 following stimulation with different wnts. The analysis indicated that Fzd6 binds to wnt3a, wnt4, wnt1, wnt5a, wnt9b, wnt10b and wnt16b. However, this technique cannot discern between direct and indirect binding, and there is the possibility that the different ligands interact with other receptors in the proximity of Fzd6.^20^

In spite these and other studies suggest an involvement of Fzd6 with the non-canonical pathway, other reports indicate otherwise. For example, it was observed that Fzd6 signalling activates β-catenin in a study of patients affected by nail dysplasia caused by germline FZD6 mutations.^21^ This study reported that wnt3a signalling causes β-catenin accumulation in healthy, but not FZD6-mutant, fibroblasts, indicating a canonical role of Fzd6 in this context. Interestingly, a recent paper revealed that Fzd6 interacts with the heterotrimeric proteins G*α*_i1_/G*α*_q_ and that the complex is dissociated following the stimulation with wnt5a.^22^ However, the G-protein-mediated signalling cascade downstream of Fzd6 and its putative role in disease and cancer remains largely obscure. Fzd6 might also mediate cellular signals through the activation of the c-Jun N-terminal Kinase (JNK), since Fzd6^high^ neuroblastoma stem cells contain higher levels of phosphorylated JNK compared to the Fzd6 negative counterparts.^23^ JNK is required for convergent extension movements that characterize gastrulation in frogs, controlled by Wnt/PCP signalling, suggesting that Fzd6 could potentially regulate PCP and JNK activation.^24^

The physiological function of FZD6 is inferred by the analysis of genetic disorders that have been linked to mutations of the locus. For instance, there is a strong correlation between frameshift mutations of FZD6 and neural tube defects, such as failure of neural tube closure along the entire body axis, suggesting the importance of Fzd6 in directing cell migration and cell polarity during brain morphogenesis.^25^ FZD6 is also important for nail development, since homozygous frameshift mutations result in the onset of a rare form of nail dysplasia. The mutations interfere with the correct localization of the receptor at the cell membrane, resulting in reduced or abrogated signal transduction.^21^ The disease is characterized by the abnormal pattering and shape of nails, consistent with a role in the control of PCP by Fzd6.

In agreement with the observations in patients bearing FZD6 mutations, FZD6-null mice manifest a phenotype characterized by defects in cell migration and tissue polarization during organogenesis. FZD6-null mice are viable, but present a disorganized orientation of the hair follicles and defects in the shaping of claws.^21, 26^ Interestingly, studies conducted on FZD6 and FZD3 double knockout mice revealed a high level of redundancy between the receptors: only abrogation of both FZD6 and FZD3 (but not the knockout of a single receptor) caused severe embryonic defects in neural tube closure and planar orientation of hair bundles on a subset of auditory and vestibular sensory cells.^27^ Further studies on FZD6-null mice suggested that FZD6 might also be implicated in limiting the activation of platelets.^28^

In conclusion, the experimental evidence gathered so far suggests that Fzd6 is required to direct the orientation and migration of cells during organogenesis, probably via activation of PCP signalling. One could speculate that reactivation of PCP in adult tissues could contribute to the metastatic dissemination of cancer cells. In the next chapter, we will review recent findings strongly suggesting that Fzd6 does play a role in tumour development and metastasis.

## Role of Fzd6 and non-canonical WNT signalling in cancer

It is now well established that the canonical, β-catenin-dependent wnt signalling pathway plays a major role in human cancer.^29, 30, 31^ Whether the PCP and non-canonical wnt pathways are also major players in oncogenic transformation is still a matter of discussion. There is some evidence though that cancer cells hijack the WNT non-canonical signalling pathway to acquire the ability to migrate and metastasize. For example, overexpression of WNT5a in melanoma and gastric cancer causes increased cancer cell migration and metastasis, but not β-catenin activation.^32, 33^ Wnt5a effects on cell migration and invasion are dependent on the activation of protein kinase C (PKC) and other effectors of the WNT/calcium pathway.^33, 34^ Interestingly, Fzd6 has also been shown to activate the WNT/calcium pathway and PKC in human cells and in Xenopus.^16, 35^ Wnt5a can, among other receptors, bind to Fzd6 and both ligand and receptor are often overexpressed in glioblastoma, suggesting that wnt5a and Fzd6 could potentially cooperate to drive migration and invasion in these tumours.^36, 37^

Several PCP core components are aberrantly expressed in tumours, contributing to cancer cell proliferation and migration. Overexpression of VANGL-1, a key component of the PCP pathway, is associated with an increased risk of relapse in breast cancer patients and its knockdown reduces breast cancer cell migration.^38^ VANGL-2 overexpression was also linked to enhanced tumour cell proliferation and poorer prognosis in breast cancer through a mechanism that involves the JNK pathway.^39^ Notably, mutations of VANGL1, like mutations of FZD6, are associated with neural tube defects, suggesting that these two PCP effectors lie in the same pathway and their aberrations might contribute to cancer.^40^ Other core components of the PCP such as Celsr1, Prickle1, Fzd3, Fzd7, Dvl2, Dvl3 and casein kinase 1 (CK1)-ε were found upregulated in B lymphocytes of patients with chronic lymphocytic leukaemia. PCP activation in these patients predicts a worse prognosis and is mechanistically implicated in transendothelial cell migration.^41^

FZD6 is critically required for the malignant transformation of B-cells in Eμ-TCL1 mice that develop chronic lymphocytic leukaemia. FZD6 expression drastically increases during leukaemogenesis, and its key role in B-cells transformation was elegantly demonstrated by crossing Eμ-TCL1 with FZD6-null mice. Mice harbouring FZD6 null alleles develop less leukaemia and have a prolonged survival.^42^ Overall these studies suggest that core PCP molecules are not only pivotal in regulating the migration of cancer cells and the development of metastasis, but could also be required for tumourigenesis.

**Figure 3:**
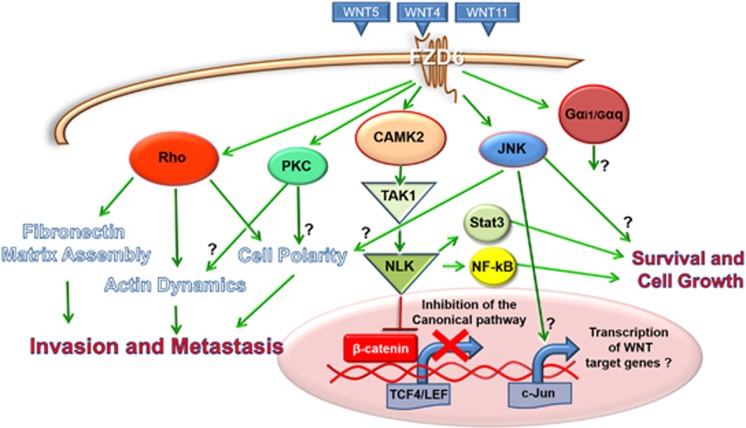
Summary of validated and putative signalling pathways downstream of Fzd6. The question marks indicate molecular functions that are only hypothetical and not fully demonstrated in previous studies.

In recent studies, it was investigated whether cues emanating from the cancer associated stroma could regulate PCP. Luga and colleagues demonstrated that exosomes secreted by cancer-associated fibroblasts can instigate breast cancer cells to acquire a motile phenotype by mobilizing PCP core components such as FZD6, DVL1, VANGL1, PK1 and stimulating the autocrine secretion of the non-canonical ligand Wnt11. Knockdown of DVL1, VANGL1, PK1 inhibited cell motility and the migratory phenotype of highly metastatic MDA-MB-231 cells, and lung metastasis were strongly reduced in mice following depletion of Pk-1.^43^ In agreement with these findings, our group has recently demonstrated that the FZD6 gene is frequently amplified and overexpressed in triple negative breast cancer, regulating the activity of the small GTPase Rho, a key component of the non-canonical wnt pathway.^44^ Rho and other members of the small GTPases family are key for orchestrating the cytoskeleton rearrangements that occur during cell migration, both in physiological and pathological conditions. Rho signalling is essential to promote cancer metastasis by regulating cell migration, extravasation and angiogenesis.^45, 46, 47^ Rho also plays a pivotal role in the PCP pathway: non-canonical wnt signalling controls the convergent extension movements through the activation of Rho and other GTPases during morphogenesis.^48^ During amphibian gastrulation, PCP activated downstream of Fzd7, Rho and wnt11 is implicated in fibronectin matrix assembly, which in turn is fundamental for the correct migration and polarization of embryonic cells during morphogenesis. Interestingly, we found that Fzd6 signalling is important for the regulation of the actin cytoskeleton and extracellular fibronectin matrix in breast cancer cells.^44^ Fibronectin is a large glycoprotein involved in connecting cells with the extracellular matrix during cell adhesion, growth and migration.^49^ Fibronectin expression is also a hallmark of Epithelial to Mesenchymal Transition, a process responsible for the metastatic behaviour of cancer cells, modulated by wnt signalling.^50^ Fibronectin is also implicated in the metastatic dissemination of cancer cells to the bones and lungs of rodents^51, 52^ and its expression inversely correlates with survival in breast cancer patients.^53^ Thus, activation of the PCP pathway through gene amplification and overexpression of FZD6 could explain, at least in part, the high metastatic propensity and the organ tropism of triple negative breast cancer cells. Indeed, RNAi knockdown of FZD6 in MDA-MB-231 triple negative breast cancer cells inhibited their motility and caused reduction of bones and liver metastases, suggesting that activation of the PCP pathway is critically important for metastatic dissemination and tumour tropism *in vivo*.^44^ A summary of the validated and putative signalling pathways downstream of Fzd6 is shown in Figure 3. FZD6 could also play a role in other forms of human cancer since increased expression has also been detected in liver, prostate, colorectal and squamous cell carcinomas (Table 1)^54, 55, 56, 57^.

## Fzd6 and cancer stem cells

Cancer stem cells are thought to derive from normal counterparts harbouring oncogenic mutations or transformed epithelial cells that underwent epithelial to mesenchymal transition and acquired a stem-like phenotype.^58^ In a previous study, our group has demonstrated that the Fzd6 receptor is expressed on the surface of stem-like cells in neuroblastomas. Fzd6^high^ neuroblastoma cells expressed high levels of mesenchymal markers such as Notch1and Twist1, formed neurospheres with high efficiency in liquid culture, were drug resistant and highly metastatic when injected orthotopically into immunodeficient mice.^23^ These biological features could be dependent on JNK activity since it is known that this kinase regulates stem cell functions through activation of the transcription factor AP-1 and the PCP pathway.^24, 59^ A further indication of the critical role played by Fzd6 in regulating cell stemness is provided by a recent study in which Fzd6 was shown to control the balance between self-renewal and survival of hematopoietic stem cells. Ablation of FZD6 strongly inhibited the repopulation of the hematopoietic compartment in the bone marrow after irradiation and transplant of donor cells in mice.^60^ It is intriguing to speculate that deregulation of Fzd6 signalling in cancer stem cells might promote uncontrolled self-renewal, homing and survival of metastatic cells in secondary organs. In agreement with this hypothesis, FZD6 is highly expressed in brain tumours containing mesenchymal, stem-like glioblastoma cells. Patients with FZD6^high^ mesenchymal glioblastomas have a higher risk of recurrence and worse prognosis compared with other glioblastoma molecular and cellular subtypes.^61, 62^ Ectopic expression of FZD6 in proneural glioblastoma cells induced the expression of stem cell markers and resulted in increased cell proliferation and sphere formation *in vitro* and *in vivo*. Conversely, ablation of FZD6 expression decreased proliferation of mesenchymal glioblastoma cells.^62^ Fzd6 controls glioblastoma cell survival and proliferation through activation of the wnt/calcium pathway, Nf-kB and Stat3, which are known to promote a mesenchymal, stem-like state and aggressive behaviour of glioblastoma cells.^62, 63, 64^

## FZD6 as a potential prognostic marker in cancer

Biomarkers are important as diagnostic tools, to predict disease outcome and ultimately direct clinical decisions and therapeutic strategies.^65^ An emblematic biomarker in oncology is HER2, whose expression is critical in determining therapeutic strategies in breast cancer.^66^ It would be important to identify new biomarkers that predicted tumour recurrence. This would benefit patients by allowing to discriminate those requiring aggressive treatments from patients who can be spared chemo- or radio-therapy.^67^ The identification of reliable markers to calculate the risk of metastatic recurrence would be particularly desirable, since metastasis is the primary cause of death in cancer patients. Tumour relapse occurs when dormant cancer cells are reactivated after months or years after chemotherapy and cancer stem cells markers have been proposed to be good prognostic indicators for tumour recurrence and metastasis.^68, 69, 70^ Cancer stem cells express membrane transporters that promote the active excretion of cytotoxic drugs and are resistant to drug killing, therefore are likely to constitute the bulk of the dormant tumour cell population.^71, 72^ In agreement with the hypothesis that FZD6 could be a cancer stem cell marker, high levels of FZD6 mRNA in neuroblastoma tumours indicate poor patient prognosis and in lymph node negative breast cancer high FZD6 expression predicts local and distant tumour relapse.^23, 44^ Positive Fzd6 immunostaining is correlated with lower distant relapse-free survival of triple negative breast cancer patients.^23, 44^ FZD6 expression is also of prognostic value in glioblastoma. Low expression of the transcription factor TCF4 coupled to high levels of FZD6 predict low survival of glioblastoma patients.^62^ These findings suggest that expression of FZD6, like other stem cell markers, could be potentially used as a prognostic biomarker in different types of human cancer.

## Concluding remarks

Despite huge research efforts, no drugs targeting the wnt signalling pathway have been yet approved for clinical use, but clinical trials are ongoing in which new molecules targeting the wnt pathway are being tested.^73^ For example, the monoclonal antibody vantictumab is currently in phase 1 trials. The antibody binds to Fzds 1, 2, 5, 7 and 8, and has been shown to reduce activation of canonical wnt signalling in preclinical cancer models.^74^ One could envisage that a naked or toxin conjugated monoclonal antibody targeting Fzd6 could be developed to treat cancers bearing high Fzd6 expression levels. In principle, such antibody could be used to deplete rare subpopulations of drug resistant, metastatic cancer stem cells within the tumour. The phenotype of mice and humans carrying genetic inactivation and/or mutation of Fzd6 is mild, mainly manifesting in the form of defects in nail and hair development.^21, 26^ Thus, it could be predicted that antibodies targeting Fzd6 should have a good therapeutic potential and be well tolerated, without affecting the function of normal cells and tissues. Time will tell whether Fzd6 targeting has a future in cancer therapeutics.

## Figures and Tables

**Table 1:** Summary of the studies in which increased expression of Frizzled 6 was detected in different cancers

*Tumour type*	*Activation mechanism*	*Phenotype*	*Reference*
Breast cancer	Gene amplification, overexpression	Increased invasion and metastasis, predicts worse prognosis in patients	^44^
Mesenchymal glioblastoma	Overexpression	Increased tumour growth in mice xenografts, predicts worse prognosis in patients	^62^
Neuroblastoma	Expression in cancer stem cells	Drug resistance, increased tumour growth in mice xenografts, worse prognosis in patients	^23^
Chronic lymphocytic leukaemia	Progressive upregulation during CD5(+) B cells leukaemogenesis	Reduced survival in mice	^42^
Hepatocellular carcinoma	Overexpression	High expression associated with lower tumour differentiation	^56^
Prostate cancer	Overexpression	Not determined	^54^
Squamous cell carcinoma	Overexpression	Not determined	^57^
Colorectal cancer	Overexpression	Not determined	^55^
